# Association between spirituality/religiousness and quality of life among healthy adults: a systematic review

**DOI:** 10.1186/s12955-021-01878-7

**Published:** 2021-10-21

**Authors:** Cezimar Correia Borges, Patrícia Roberta dos Santos, Polissandro Mortoza Alves, Renata Custódio Maciel Borges, Giancarlo Lucchetti, Maria Alves Barbosa, Celmo Celeno Porto, Marcos Rassi Fernandes

**Affiliations:** 1 Faculdade de Medicina, Programa Ciências da Saúde, Universidade Federal de Goiás, Secretaria – 1ª Av. s/n – Setor Universitário, Goiânia, Goiás CEP: 74605-020 Brazil; 2 Unicerrado, Centro Universitário de Goiatuba, Rod. GO-320 s/n – Jardim Santa Paula, Goiatuba, Goiás CEP: 75600-000 Brazil; 3 Universidade Estadual de Goiás, Unidade Universitária de Itumbiara, Av. Modesto de Carvalho s/n – Distrito Agroindustrial, Itumbiara, Goiás Brazil; 4 Faculdade de Medicina, Universidade Federal de Juiz de Fora, Av. Eugênio do Nascimento, s/n° - Dom Bosco, Juiz de Fora, Minas Gerais CEP: 36038-330 Brazil

**Keywords:** Spirituality, Religion, Quality of life, Set, Psychology, Adult

## Abstract

**Background:**

Health-related quality of life (HRQoL) is determined by multiple factors that include components such as spirituality and religiousness (S/R). Even though various systematic reviews have investigated the association between S/R and improved health outcomes in the most different groups, healthy young individuals are seldom addressed.

**Objective:**

To assess the association between S/R and HRQoL among young, healthy individuals.

**Methods:**

Systematic review of papers published in the last ten years and indexed in four academic research databases (PubMed, Web of Science, Cochrane Library, and Scopus) and two gray literature databases. Inclusion criteria were studies assessing S/R and HRQoL using validated instruments and assessing healthy adults (i.e., non-clinical patients, not belonging to any specific group of chronic diseases), aged between 18 and 64 years old.

**Results:**

Ten out of 1,952 studies met the inclusion criteria: nine cross-sectional and one longitudinal cohort study, in which 89% of the participants were college students. Nine studies report a positive association between S/R and HRQoL, while one study did not report any significant association. The main HRQoL domains associated with S/R were the psychological, social relationships, and environment domains, while the S/R most influent facets/components were optimism, inner strength, peace, high control, hope, and happiness.

**Conclusions:**

Higher S/R levels among healthy adult individuals were associated with higher HRQoL levels, suggesting the S/R can be an important strategy to deal with adverse environmental situations even among those without chronic diseases, enhancing the wellbeing of individuals.

*Registration of systematic review*: PROSPERO—CRD42018104047.

## Background

Today’s society is increasingly concerned with aspects that influence the quality of life (QoL) of different populations, youth, adults, or the elderly. QoL can be addressed by different fields of knowledge, such as social, political, economic, or the health field [1, 2].

In the health field and academic milieu, QoL has been associated with a more comprehensive concept, which does not necessarily refer to a lack of disorders or health problems. In this context, health-related quality of life focuses on the individuals’ subjective perception of general health concerning domains/components of physical and mental/psychological health, social relationships, and environment [1, 2].

Even though the physical and mental health components are the most frequently investigated in scientific studies addressing QoL, other important factors have been recently addressed, such as satisfaction, quality of relationships, personal fulfillment, wellbeing, access to cultural and religious events, freedom, and leisure, among others [3, 4].

Based on the assumption that physical, psychological-mental, and social aspects determine an individual’s QoL, personal beliefs and spirituality/religiousness (S/R) levels are important variables to be considered in QoL’s global construct [5].

The way individuals relate to everyday stressful and adverse situations, that is, their coping strategies, tend to influence their perceived QoL directly, while S/R involvement, also known as spiritual/religious coping (SRC), is among the factors that determine coping strategies. Studies report a positive correlation between positive SRC and improved QoL, as well as an inverse correlation when this strategy is negatively used (e.g., God punishment, religious conflicts) [6–9].

Although there is apparent overlapping, religiosity and spirituality are not necessarily synonymous. Koenig [10] notes that religiosity is linked with an individual’s participation in an organized system of beliefs, rituals, and symbols to access the sacred (God, Higher Power). On the other hand, spirituality is characterized as a personal search for comprehensive answers to existential questions, the meaning and relationship with the sacred or transcendent that may, or may not, include involvement with religious practices or a specific religion or religious community. Thus, a spiritual individual may not be affiliated with a specific religion [6, 7].

Authors addressing this subject report difficulties establishing a definition or concept of S/R, which results in different perspectives of the instruments designed to measure these variables. Note that spirituality has been historically conceptualized under two general approaches. The theistic approach (based on God's existence or a higher power) and the non-theistic approach based on existential, humanistic, and secular elements [11–16].

Most studies addressing the relationship between S/R and QoL address individuals affected by different pathologies, such as neoplasms [17], HIV, cardiovascular diseases, or neurological/psychiatric disorders [10, 18, 19]. Similarly, various studies have addressed elderly individuals [23–26] or caregivers susceptible to burnout [20–22].

Some reviews [10, 17, 25, 26] show many studies reporting positive associations between S/R with QoL among individuals with severe diseases, suggesting that these populations’ quality of life is significantly benefited from S/R components, considering the greater fragility or vulnerability to which they are exposed during the disease process and often invasive treatments, as is the case of cancer patients [17]. Nevertheless, there is a gap in the scientific literature regarding studies addressing the association between S/R and QoL among healthy individuals, that is, individuals presenting no comorbidities. Even though previous studies have addressed this association [27–30], to our knowledge, no systematic reviews have compiled data to identify evidence on this topic. Understanding S/R among healthy individuals is relevant because the positive effects of these variables on QoL parameters previously found among other groups are also expected in this population. Thus, S/R is a relevant complementary strategy to promote health, considering the numerous challenges typically imposed on the routine of communities worldwide.

In this sense, this study’s objective was to investigate how S/R is associated with the QoL among healthy adult individuals based on a systematic literature review. Specific objectives include identifying the main components (psychological, physical, social relationships, and environment) associated with QoL in this population.

## Method

This systematic review was developed according to recommendations provided by PRISMA—Preferred Reporting Items for Systematic Reviews and Meta-Analysis [31], and was registered in the International Prospective Register of Systematic Review (PROSPERO) under No. CRD42018104047 [32].

### Eligibility criteria

This study’s objectives were based on the adapted PICOS tool:

- P = Population: healthy adult individuals
- I = Exposure/Intervention: spirituality/religiousness variables and personal beliefs;
- C = Comparison: Comparison between exposed and non-exposed groups;
- O = Outcome; Health-related quality of life;
- S = Study design: observational or intervention studies.

Inclusion criteria were:

- Samples composed of individuals aged between 18 and 64 years old and not considered chronic patients or ill individuals [33];
- Studies with observational descriptive, cross-sectional, prospective designs and experimental trials;
- Associations between S/R and QoL were assessed using quantitative instruments. All dimensions of QoL were included in the analysis (i.e., general, psychological, physical, environmental, and social dimensions)

Exclusion criteria were:

- Samples composed of children or adolescents (younger than 18 years old) or elderly individuals (65 years old or older);
- Studies addressing patients or individuals with physical or mental conditions [33] or caregivers of individuals experiencing these health conditions [34];
- Studies with a qualitative approach or not using cross-culturally validated and reliable quantitative instruments and/or renowned measures used in the scientific milieu to assess S/R and QoL.

### Search strategies

The terms used to search for primary studies were based on MeSH descriptors (Medical Subject Headings) and applied in the following databases: PubMed, Web of Science, Cochrane Library, and Scopus, in addition to two gray literature databases, Brazilian Digital Library of Theses and Dissertations (BDTD) and OPEN GREY (Grey literature in Europe). The references of the studies selected were also manually consulted. The general terms used were: “spirituality”, “religions”, “quality of life” and “health-related quality of life”. These terms (written in English, Portuguese, and Spanish) were applied in the advanced search systems according to the resources available in the different databases. Afterward, the search was refined using the Boolean operator “NOT” for patients, disease, caregivers, children, adolescents, and elderly. Table 1 presents the strategies used in the different databases.

**Table 1:** Description of search strategies and results according to each database

Database	Combined search terms	Boolean operators
	OR/AND	NOT/AND NOT
PubMed	(“spirituality” [Title/Abstract]) OR (“religions” [Title/Abstract]) AND (“quality of life” [Title/Abstract]) OR (“health-related quality of life” [Title/Abstract])	NOT (“patients” [Title/Abstract]) NOT (“disease” [Title/Abstract]) NOT (“caregivers” [Title/Abstract]) NOT (“children” [Title/Abstract]) NOT (“adolescent” [Title/Abstract]), NOT (“elderly” [Title/Abstract])
Web of science	TÓPICO: (“spirituality”) OR TÓPICO; (“religions”) AND TÓPICO: (“quality of life”) OR TÓPICO: (“health-related quality of life”)	NOT TS = “patients”; NOT TS = “disease;” NOT TS = “caregivers”; NOT TS = “children” NOT TS = “adolescent”; NOT TS = “elderly”
Scopus	(TITLE-ABS-KEY (“spirituality”) OR (TITLE-ABS-KEY (“religions”) AND (TITLE-ABS-KEY (“quality of life”) OR (TITLE-ABS-KEY (“health-related quality of life”)	AND NOT “patients”; AND NOT “disease;” AND NOT “caregivers”; AND NOT “children” AND NOT “adolescent”; AND NOT “elderly”
Cochrane	(“spirituality”): ti,ab,kw OR (“religions”): ti,ab,kw AND (“quality of life”): ti,ab,kw OR (“health-related quality of life”): ti,ab,kw	AND NOT “patients”; AND NOT “disease;” AND NOT “caregivers”; AND NOT “children” AND NOT “adolescent”; AND NOT “elderly”
BDTD	“espiritualidade” All fields; “religiosidade” All fields; “qualidade de vida” All fields; “qualidade de vida relacionada à saúde Todos os campos	Not applicable
Open grey	“spirituality” AND “quality of life”	Not applicable

After the electronic search, the titles and abstracts were read, and when the information was not sufficiently clarified in the title or abstract, the method session was also read. Finally, the papers’ full texts were read, and the references were consulted to identify potentially eligible papers.

### Data extraction

The studies’ eligibility was determined using a data extraction form addressing the characteristics of samples (population), studied variables (dependent and independent), and the methods employed to assess S/R and QoL, including criteria for relevance test I and II (“Appendix 1”).

Two researchers independently searched and extracted data, selecting and electing the studies that were coherent with the study criteria. Inter-rater reliability was measured by the Kappa statistic [35] using SPSS 21.0.

### Risk of bias

The studies' quality was assessed using two instruments: Critical Appraisal Checklist For Analytical Cross-Sectional Studies and Critical Appraisal Checklist For Cohort Studies [36]. These instruments are intended to assess potential bias, such as selection, performance, detection, and attrition bias.

These checklists present the items that should be verified in each review. Four possible answers are provided to each question: “yes” (when the requirement is met), “no”, “unclear”, or “Not applicable”. The number of items differs between the two instruments. Eight questions are asked to assess cross-sectional studies and 11 to assess longitudinal studies.

Cut-off points for the number of items checked (percentage) were the criterion determining the quality of each study:

- High-quality study (low-risk bias) = 80% to 100% of “yes” answers;
- Moderate-quality study (moderate-risk bias) = 60% to 79% of “yes” answers”;
- Low-quality study (high-risk bias) ≤ 60% of “yes answers”.

## Results

**Fig. 1:**
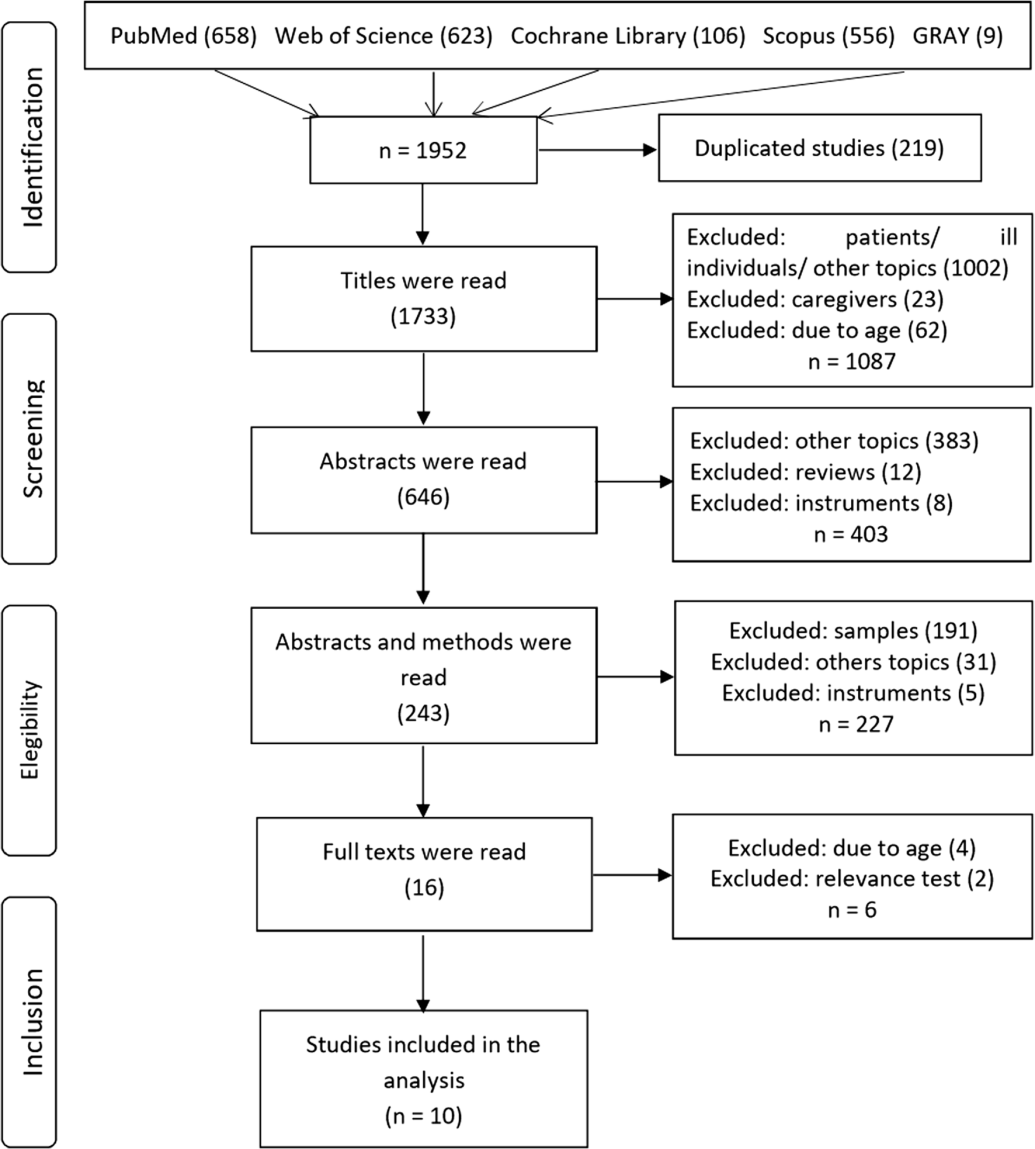
Study selection and selection criteria flowchart

As shown in Fig. 1 (PRISMA Flow Diagram), 1.952 records were identified in the initial search from the four scientific periodical databases and two gray literature sources. Of these, 1.936 papers were excluded due to duplicate versions or for not meeting this review’s objectives; that is, studies addressed patients, caregivers, elderly individuals, or children and adolescents; the topic was unrelated to this study’s objectives; or were literature reviews. In addition to the complementary search performed in the studies’ references, the full texts of 16 papers remained. After reading the full texts, six papers were excluded for addressing mixed samples, elderly individuals or minors, or for not meeting the inclusion criteria. One of the papers was excluded because the participants’ age was not reported [5], though the author kindly provided information via email. In the end, ten papers were included to be thoroughly analyzed.

The level of agreement between the two reviewers regarding the studies’ eligibility was verified using the Kappa coefficient, which was 0.81, that is, significant (*p* < 0.05) high agreement was obtained according to Landis and Koch [35].

Table 2 presents a synthesis of the ten papers included in this review, with information concerning the studies’ designs.

**Table 2:** Assessment of studies quality

Studies	Questions											Total (%)	Risk of bias
	1	2	3	4	5	6	7	8	9	10	11		
Chai et al. [41]	Y	Y	Y	Y	Y	Y	Y	Y	–	–	–	100	Low
Hsu et al. [37]	N	Y	Y	Y	Y	N	Y	Y	–	–	–	75	Moderate
Krägeloh et al. [30]	Y	Y	Y	Y	N	N	Y	Y	–	–	–	75	Moderate
Deb and Strodl [29]	Y	Y	Y	Y	Y	Y	Y	Y	–	–	–	100	Low
Felicilda-Reynaldo et al. [42]	Y	Y	Y	Y	Y	Y	Y	Y	–	–	–	100	Low
Lau et al. [43]	Y	Y	Y	Y	Y	Y	Y	Y	U	U	Y	82	Low
Pillay et al. [28]	N	Y	Y	Y	Y	Y	Y	Y	–	–	–	87	Low
Casu et al. [38]	Y	Y	Y	Y	Y	Y	Y	Y	–	–	–	100	Low
Dadkhahtehran et al. [39]	Y	Y	Y	Y	Y	Y	Y	Y	–	–	–	100	Low
Gonçalves et al. [40]	Y	Y	Y	Y	Y	Y	Y	Y	–	–	–	100	Low

Y: Yes, N: no, U: unclear

Table 2.

The main results are presented below:

### Population

A total of 4.337 individuals composed the samples of the ten studies selected. Only one study, by Hsu et al. [37], does not report the distribution between men and women. The corresponding author confirmed the lack of this information via email. Based on the remaining papers’ samples, a more significant proportion of women (55.23%) compared to men (44.77%) were addressed in the studies.

College students were the most frequently addressed (n = 3.860), representing 89% of all the participants and composing the sample of seven of the ten studies included. Of these, 30.15% belonged to programs in the health field (medicine and nursing), while the remaining participants were from different unreported fields of knowledge.

In addition to young students, two studies involved infertile couples [38, 39], and another involved a population of individuals living in a riverside community in Pantanal, Brazil [40]. The average age (34.66 years old) in these studies was higher than that of college students (22.05 years old).

As for the studies’ countries of origin, New Zealand was the country with most studies, three papers [30, 37, 41], followed by Brazil with two papers [38, 40], and the remaining studies were conducted in India [29], the United States [42], China [43], South Africa [28], and Iran [39].

### Study designs

All were observational studies, and nine had characteristics of cross-sectional descriptive studies, and one was a prospective longitudinal cohort study [43] with a three-year follow-up.

### Objectives of the studies

The studies’ primary objectives focused on the association between S/R and QoL parameters, which meet this review’s objectives. The secondary objectives frequently reported were investigating S/R’s role in mental health aspects such as stress, anxiety, depression level, and coping strategies.

### Instruments used to assess S/R

There are various instruments available in the literature to assess S/R, some primarily address spirituality, others address religiousness, while some apply to both. Three studies [29, 40, 42] adopted the Duke University Religion Index (DUREL), while the WHOQOL Spirituality, Religiousness, and Personal Beliefs (SRPB) was also adopted by three studies [30, 37, 41]. Four studies adopted instruments composed of dimensions or scales intended to assess spirituality or personal beliefs [28, 38, 39, 43]. Eight studies asked the participants to report religious adherence or affiliation [28–30, 37, 40–43]. Five of these studies reported that between 27 and 76.7% of the participants had no connection with any specific religion. Among those who reported religious affiliation, most were adept to Christianity, except for the study conducted in India [29], where Hinduism was most frequently reported (78.1%).

### Instruments used to assess QoL

The WHOQOL-bref was widely used to assess QoL; eight of the ten studies adopted it [28–30, 37, 38, 41–43]. Two studies adopted the Short Form Health Survey—SF-36 [39], and SF-12 [40]. All the QoL instruments were cross-culturally validated for different languages, were considered generic and related to health in general, and not necessarily to some clinical condition or a specific age range; hence these are appropriate for samples of healthy adults [2]. The instruments contain domains/components based on the health concepts most frequently disseminated worldwide, encompassing physical, psychological, social, and environmental aspects [44].

### Results

The ten studies included in this review intended to investigate the effect of S/R as a predictor variable (independent) on QoL (and its domains), the health-related outcome variable (dependent). The statistical models most frequently employed for this purpose were multiple linear regression analyses, coefficient of correlations and determination, and covariance measures.

The findings were very similar among most of the studies. Nine papers report a positive association between S/R levels and QoL outcomes; the only exception was the study conducted by Gonçalves and collaborators [40] in which the authors report no influence of S/R on QoL measures, though a positive correlation was found between anxiety and depression based on intrinsic and non-organizational religious S/R.

Individuals with greater religious involvement scored higher in spirituality facets and personal beliefs. However, the factor that determined QoL the most was not having a direct relationship with any specific religion, but the spirituality/personal beliefs the participants adopted. Note that higher spirituality scores were directly associated with improved QoL, even for those without a formal religious affiliation, emphasizing mental QoL with its domains/components and environmental relationships [28, 29, 41–43].

The QoL domains/components that were positively influenced by S/R standards the most were psychological aspects (70% of the studies), followed by the social relationships and environment domains (20% each), with the least change being on the physical domain (10% of the studies).

Among the facets/components of spirituality and personal beliefs that most determined QoL outcomes were hope, optimism, the meaning of life, inner peace, wholeness and integration, spiritual strength, faith, and high self-control [29, 30, 37].

The positive effects of S/R on mental health parameters, both on the QoL domains and stress and depression, were highlighted in all the papers selected, and for the most frequent population, that of college students, the S/R strategies were very relevant in the QoL final result.

### Study quality and risk of bias

Table 2 presents the results concerning the studies' quality based on the two checklists and cut-off points adopted to classify the risk of bias. Six studies positively answered 100% of the questions, and two studies answered 82% and 87% of the questions. Thus, most studies (8 out of 10) present high quality, i.e., low risk of bias. The other two studies were considered to present moderate quality (75% of risk of bias). The assessment of risk of bias indicates that most studies established appropriate selection criteria, properly described the individuals in the samples, the exposure measurement was valid and reliable, and confounding factors were treated with appropriate statistical models.

## Discussion

This systematic review shows that all the studies included, except for one, showed a positive association between S/R and QoL among healthy adults. The main components of these associations were psychological, followed by social relationships and environment. In this sense, evidence indicates that S/R plays a relevant role in promoting health and wellbeing in this population (Table 3).

**Table 3:** Synthesis of the studies distributed according to similar instruments measuring S/R and QoL

Author/year /study design/country	Objectives	Methods		Results	Conclusions
		Sample	S/R and QoL instruments		
Chai et al. (2012) [41] Observational cross-sectional New Zealand	To verify whether domestic and international students, with different ethnicities, adopt S/R coping strategies and how these affects QoL	679 college students Female (495), Male (184) Average age = 22.59 years old	WHOQOL-SRPB; WHOQOL-bref	Europeans x Asians: SRPB = 24.79 and *26.85 Coping with stressors F(1.433) = *30.81, higher among Asian students S/R predicts psychological domains among Asian students (β = *0.22)	↑ SRPB»► ↑ ability to cope with stress; ↑ SRPB»► ↑ psychological domains, social relationships, especially among Asian students when compared to European students
Hsu et al. (2009) [37] Observational cross-sectional New Zealand	To verify whether S/R is related with QoL and with coping mechanisms among domestic and international students	382 college students Gender not reported Average age = 23.78 years old	WHOQOL-SRPB; WHOQOL- bref	Domestic x international students: SRPB = 24.01 and 26.92* Correlations SRPB x psychological domain = *0.36 and *0.32 SRPB x social relation domains = 0.13 and *0.23	↑ SRPB»► ↑ psychological domain in both groups; ↑ social relationships among internationals ↑ SRPB»► ↑ social relationships among international students
Krageloh et al. [30] (2015) Observational cross-sectional New Zealand	To verify the effect of S/R and personal beliefs on the QoL of student affiliated with a religion or not	275 medical students Female (156), Male (118) Average age = 22.86 years old	WHOQOL-SRPB; WHOQOL- bref	General mean of the WHOOL-SRPB facets: *14.35 (religious), 10.82 (non-religious)	↑ SRPB»► ↑ General QoL ↑ hope, optimism, and meaning of life»► ↑ psychological domain for all students
Deb and Strodl [29] (2018) Observational cross-sectional India	To verify association between S/R and QoL among graduate Indian students	475 graduate students Female (234), Male (241) Average age = 22.13	SAI: DUREL, EWBS, NRCOPE, MHLC; WHOQOL-bref	Most significant correlations: NRCOPE x psychological domain = *0.60 NRCOPE x QV = *0.60 Most significant regressions: All S/R measures with psychological domains (F = *35.10) and Environment (F = *25.04)	↑ E/R»► ↑ psychological and environmental domains ↑ E/R»► ↑ Total QoL, emphasis on the hope and high self-control facets
Felicilda-Reynaldo et al. (2019) [42] Observational cross-sectional USA	To verify the predictive role of S/R on the QoL of nursing students from four countries	659 nursing students Female (112), Male (547) Average age = 21.14 years old	DUREL, SCS; WHOQOL- bref	Linear regression S/R x QoL: Organizational and non-organizational religiosity x physical domain (β = *0.98 and β = *0,99), physical domain (β = *0.98 and β = *0.99). Non religious coping x psychological domain (β = *0.98)	↑ Organizational and on non-organizational S/R»► ↑ physical domain and environment ↑ Non-organizational S/R»► ↑ psychological domain; ↑ Non-religious coping»►↑ QoL in the 4 domains
Lau et al. (2015) [43] Longitudinal observational China	To verify the causal model in the relationship between spirituality and QoL during three years among college students	1160 college students Female (787), Male (373) Average age = 20.9 years old	STS; WHOQOL-bref	Direct effects between the 1^st^ and 3^rd^ assessment of the STS in WHOQOL-brief (β = *0.130). The inverse did not occur (β = 0.098)	Spirituality predicted QoL but not vice-versa, regardless of affiliation or gender
Pillay et al. (2016) [28] Observational cross-sectional South Africa	To assess the role of spirituality in the relationship with depressive symptoms and QoL among medical students	230 medical students 166) Average age = 21 years old	SIBS; WHOQOL-bref	r de person SIBS x WHOQOL-brief = *0.294 SIBS x depression symptoms = * − 0.143	↓ S/R»► ↑ depressive symptoms ↑ S/R»► ↑ QoL
Casu et al. (2018) [38] Observational cross-sectional Brazil	To verify how spirituality (S) is associated with stress and QoL among infertile couples	152 couples (n = 304) Average age = 36.67 years old	FACIT-sp; WHOQOL-bref	Total effect of spirituality on QoL, B = * 0.55	↑ S was positively associated with ↑ individual QoL ↑ S ↓ stress among infertile couples
DadkhahtehranI et al. (2018) [39] Observational cross-sectional Iran	To assess the association between religious coping and QoL among infertile individuals and their spouses	200 couples (n = 400) Average age = 30.71 years old	Brief RCOPE scale; SF-36	Positive religious coping among the wives x QoL (β = *0.51), negative coping x QoL (β = * − 1.31). Positive coping among the husbands x QoL (β = *0.80)	Personal beliefs positively influence QoL, but only individually among the wives; this effect was not found among their partners
Gonçalves et al. (2018) [40] Observational cross-sectional Brazil	To investigate the relationship between S/R and mental health and QoL in a riverside community	129 individuals Female (59), Male (70) Average age = 36.6 years old	SSRS, DUREL; SF-12	Linear regression S x anxiety (β = * − 0.236) and depression (β = * − 0.398). Non-organizational religiosity x anxiety (β = * − 0.250), x depression (β = * − 0.351). Intrinsic religiousness x depression (β = * − 0.315)	↑ S»► ↓ anxiety and depression symptoms; ↑ non-organizational R and intrinsic R»► ↓ anxiety and depression; S/R measures were not associated with QoL

DUREL (Duke University Religious Index), EWBS (Existential Well-Being Scale), FACIT-Sp (The Functional Assessment of Chronic Illness Therapy–Spiritual Well-Being Scale), MHLC (Multidimensional Health Locus of Control Scale), NRCOPE (Negative Religious Cope), RCOPE (Religious Coping Strategies), SAI (Spiritual Attitude Inventory) SIBS (Spiritual Involvement and Beliefs Scale), SSRS (Spirituality Self Rating Scale), STS (Spiritual Transcendence Scale), SCS (Spiritual Coping Strategy Scale), SWBQ-G (Spiritual Well-Being Questionnaire Germany), SWBS (Spiritual Well-Being Scale) WHOQOL-SRPB (World Health Organization Quality of Life**—**Spirituality, Religiousness and Personal Beliefs)

(*): considered significant (*p* < 0.05 or < 0.01)

(↑): Increase, (↓): decrease, (»►): resulted/produced/meant

These results corroborate previous reviews reporting this same association among individuals with specific diseases or experiencing conditions that complicate physical and mental health [25, 26, 45]. However, this review adds to the scientific literature, showing that S/R may be relevant among individuals without prior diseases, such as the ones assessed in this study.

Considering that healthy individuals were assessed, most studies addressed college students. Even though college students are healthy from the perspective of an absence of chronic diseases, in general, these individuals are in a phase of transition in many spheres of life, facing numerous challenges while surrounding by unfamiliar people, and having to dedicate many hours to independent studying, often experiencing many doubts and uncertainty regarding their academic and professional lives, which predispose these individuals to health conditions, especially mental problems [46, 47]. These daily demands may impact the QoL of individuals in a college context, and S/R seems to work as an important coping mechanism, as the studies included in this review suggest [29, 30, 37, 41–43]. Additionally, other studies addressing infertile couples also report similar results [38, 39].

Among the QoL components most frequently associated with S/R, the psychological component was the most important. The most significant evidence available in the S/R field is related to the psychological dimension and mental health [5, 19, 25, 48]. Nine of the studies in this review report positive associations between S/R with mental health and/or psychological parameters. Six studies [28–30, 37, 41, 42] reported that the highest scores were obtained in the psychological domain of QoL, in addition to decreased stress [38, 41], anxiety and depression [28, 40].

Two studies report positive correlations between S/R and improved psychological QoL among college students in New Zealand. The first study [41] emphasizes that religious coping was determinant among international students of Asian ethnicity, comparing to European students. The second study [37] reports this same association between domestic and international students, though the latter scored higher on the SRPB. Thus, international students, especially those of Asian ethnicity, presented greater religious involvement than the domestic and European college students and presented greater psychological QoL.

The study by Krageloh et al. [30] addressed medical students and also reports that religious affiliation favored higher scores on the SRPB. However, both religious and non-religious individuals showed positive associations between S/R and psychological QoL in terms of hope, optimism, and meaning of life. Considering that spirituality facets predict QoL, the study addressing graduate students show that existential wellbeing was determinant in psychological QoL, while hope and high self-control influenced total QoL, though not religiousness per se [29].

As previously mentioned, college students are exposed to environmental conditions that may lead to important psychological disorders such as having to face an intense study routine in a country other than their country of origin, dealing with cultural, communication, habits, weather, and other aspects that make them vulnerable to emotional and mental conditions [46, 47]. It is known that medical students typically face a challenging study routine and deal with stressful situations during clinical and hospital practice. Thus, the studies previously mentioned, together with those included in this review, show that increased S/R levels are positively correlated with improved psychological QoL among students [29, 30, 37, 41–43].

Note that there is a clear overlap between psychological QoL and mental disorders and four of the studies included in this review found positive associations between S/R and depression and stress [28, 38, 40, 41]. In this line, other studies addressing mixed samples of adults and elderly individuals, healthy and unhealthy people, also report a relationship between higher levels of S/R with improved psychological QoL and decreased stress and depression, according to the influence of facets such as peace, meaning, optimism and happiness [5, 48].

Analyzing the physical health component related to QoL from the notion that mental and physical health are inextricably linked, there is a natural expectation that positive correlations between S/R and psychological QoL also improve physical QoL. This has been discussed by some authors, especially those addressing individuals under complicating health conditions, outlining clinical applications of these strategies with patients [10, 19, 26, 48] based on robust data from observational studies and clinical trials including cancer patients, individuals with HIV, heart conditions, or trauma, among others [10, 45, 49].

As far as we know, there are no studies separately assessing the influence of S/R on QoL physical aspects because these are observed together with the remaining domains. However, there seems to exist a consensus that physical QoL outcomes are not as significant as mental health QoL outcomes. Indirect effects on relevant behavior are more frequently reported, such as greater pain tolerance, greater vitality (energy), and less binge eating, among others [10, 45, 49]. The most significant concern around the connection between mental and physical health concerns how psychological disorders affect physical health, considering that negative emotions lead to physiological disorders in the body systems, poorer adherence to treatments, lower life expectancy, and worsening of QoL as a whole [10, 19, 26].

Considering that the studies’ samples were primarily composed of college students, the physical domain was the least evident, as young individuals with this profile are usually in good physical conditions. Only one study presented a positive correlation between S/R and scores in the physical domain [42]. This association was similar among nursing students between organizational and non-organizational religiosity.

As for the remaining components of QoL, social relationships and environment play a vital role in individual and collective health constructs, elements connected with the social relationships, and environment domains of QoL. As confirmed by previous studies [10, 19, 25], S/R variables are useful in this relationship.

Both the studies addressing college students from New Zealand report that higher levels of S/R were correlated with improved outcomes in the social relationships domain as well as greater ability to handle stress [37, 41]. As for international students, giving continuity to the culture they learned at their country of origin and remaining involved with organizational religiosity, as was the case of Asian students, resulted in improved QoL compared to European students, which was certainly influenced by greater interaction with other people and, consequently, more significant social support.

Loneliness linked with a lack of social support has been found among college students. This is of concern because loneliness often leads to mental health disorders such as severe depression and even suicide in this population [47, 50, 51]. Participating in collective socializing activities, among which those linked to S/R, seem to be interesting alternatives in this context. Previous studies show that S/R may foster social interaction, mainly through religious attendance and religious support groups.

The environment domain from the WHQOL-bref involves the physical environment, including physical safety and security at home and workplace, having financial resources, accessibility to health care, transport, opportunity for recreation and leisure, and to acquire information [1, 3, 4].

Some reviews highlight that social support and environment may be dissatisfying for some groups of more vulnerable people, such as the elderly, and in this sense, S/R plays a vital role in improving QoL perception [5, 19, 25].

In this aspect, Deb and Strodl [29] reports that higher scores obtained by Indian graduate students in the WHOQOL-bref’s environment domain were associated with higher scores in the S/R facets, especially hope and high self-control, but not necessarily religiousness per se*.* Likewise, Felicilda-Reynaldo et al. [42] report that not only the environment domain but also the remaining domains of QoL were determined by higher spirituality and non-religious coping scores. S/R seems to enhance the individuals’ resiliency and tolerance to worse environmental conditions, which could help understand these findings. Therefore, the evidence found in this review supports the notion that higher S/R levels result in better QoL perception among healthy young adults without chronic diseases. Naturally, as already mentioned, there is a significant concern on the part of the scientific community in investigating the role of S/R in improving the QoL of people experiencing complicating stages of health. However, this review shows that S/R variables are also relevant for groups of individuals who are not currently experiencing any significant general health problem. Because the participants were young individuals, their total QoL presents good means precisely because of the physical domain. However, we know that the total QoL construct depends on other domains and components that may be undesirable or under threshold levels for some people.

The psychological domain of QoL is the component of greatest vulnerability among young individuals. An increased number of mental health problems is acknowledged, many of which begin even before adulthood [46, 47, 50, 51], which is of concern and constant challenge faced by parents, teachers, and health workers, who seek efficient measures to improve these conditions, not always easily identified. Unlike patients who show evident debilitating physical manifestations, psychological problems in non-patients (or “non-ill individuals”) may go unnoticed. As a result, these conditions may progress and culminate in catastrophic events, being a surprise even for families and close people [52].

Precisely the psychological domain was the most frequently mentioned by the studies included in this review. This component appears positively associated with increased levels of S/R among college students. Both intrinsic spirituality and organizational and non-organizational religiosity were fairly correlated with psychological QoL and social relationships and environment.

These individuals may perceive the many challenges imposed throughout their lives with different levels of difficulty, depending on their S/R level. In the medium and long term, S/R may be a turning point between normal health conditions and illness processes at the mental, physical, social levels, thus affecting total QoL. That is, S/R determines individual and/or collective health in the population in general.

Despite the positive association between S/R with improved QoL, there are indications that an opposite relation may occur, that is, when there is a negative religious involvement, as reported by Bonelli and Koenig [19] in a systematic review addressing 43 primary studies published from 1990 to 2010. Two (4.7%) of these studies presented a negative influence of S/R, in which it triggered mental disorders. The integrative review performed by Counted, Possamai and Meade [25] reports a proportion even greater; three (15%) of the 20 papers published between 2007 and 2017 report a negative influence of S/R on QoL from a perspective of relational spirituality, in which spirituality depends on the sacred (a God). Because the studies addressed in this review assessed S/R from a more general and positive perspective, that is, religious conflicts or negative coping were not addressed, the results presented here are positive. Further studies addressing negative relationships with religiousness are needed in this field of research.

## Limitations

Some of this review’s limitations refer to the fact that only observational studies were selected. Of these, most were cross-sectional, and only one study provided a prospective longitudinal analysis. In the scientific milieu, clinical trials are considered to provide better evidence; however, studies addressing the effects of S/R on HRQoL more frequently focus on patients while researchers in this field do not seem to be interested in addressing healthy individuals.

Social, economic, and cultural differences between countries directly influence the different communities’ religious and spiritual customs and beliefs. As a result, measuring these parameters using instruments to assess individuals’ subjective perceptions is difficult. Thus, there will always be a limitation to obtain a reliable or gold standard measure to assess these variables.

Finally, a potential limitation refers to the criteria used to include only healthy individuals. It is difficult not only to fit individuals into a broad concept of general health, but researchers do not have total control over the participants’ health; that is, one cannot be sure whether the individuals are healthy because clinically assessing the physical and mental health of a large number of voluntary participants is usually unfeasible. The studies addressed here involved hundreds of participants so that only indirect assessments using self-report questionnaires were performed.

Studies attempted to minimize these problems because young individuals are less susceptible to health complications than elderly individuals. For this reason, 65+-year-olds were excluded because there is a high prevalence of chronic diseases in this population, which would hinder the interpretation of results. Additionally, most participants were college students with an active academic life, presenting high QoL scores (emphasis on the physical domain), so we can infer that the samples were composed of healthy non-patient individuals.

## Strengths and future directions

Thus far, this is the first review addressing the association between S/R and health-related QoL among healthy adult patients, without chronic diseases, non-elderly, and non-caregivers of patients. Differences among the countries involved in the studies, representing various continents, support the possibility that the results represent populations with these characteristics worldwide.

The results show that, regardless of the various ways S/R is appropriated, all seem to contribute to improved QoL outcomes in this population, specifically the psychological, social relationships, and environment domains. Facets such as optimism, inner strength, peace, high self-control, hope, and happiness, were the most significant in this relationship, and the improvement reported by most studies in QoL indicates complementary benefits of this dynamic, such as decreased stress, anxiety, and greater ability to deal with numerous challenges in the environment.

The current global pandemic incites negative feelings, hopelessness, and psychological problems triggered by the severe consequences on the population, including younger individuals. In this context, coping strategies are essential to ensure the quality of life among healthy individuals [41–43]. Therefore, religious and spiritual beliefs seem to be strongly associated with quality of life, even among individuals without chronic diseases, suggesting improved outcomes can be obtained in times of crisis. This review supports this hypothesis, revealing that beliefs positively influence young individuals without comorbidities.

In addition to the studies presented in this review, other cross-sectional observational studies, cohort studies, and intervention studies with clinical trials, similar to those addressing other groups (e.g., patients), are needed to complement evidence and acquire a better understanding of all the mechanisms involved in the S/R and QoL relationship.

## Conclusions

The conclusion is that higher levels of spirituality and religiousness are positively associated with improved HRQoL among healthy young adults with an emphasis on the psychological, environmental, and social relationships domains. Spirituality, understood as a broad concept on how individuals assign meanings to life in a multidimensional manner, creating inner values in the face of various situations of the human condition, regardless of a specific religious affiliation, appears as the most determinant factor in this positive relationship.

Whether it is linked to a religion or not, intrinsic spirituality seems to be an interesting strategy, even for those young individuals not facing significant health complications. These constructs can benefit everyday life and even being considered a preventive factor considering the typical conditions triggering mental health disorders, such as stress, anxiety, and depression.

We suggest that observational studies addressing healthy young individuals are complemented by intervention studies, similar to clinical trials conducted with chronic, terminal patients or those under conditions that strongly alter their physical and mental conditions, to support the findings reported in this review.

## Data Availability

The set of data collected and analysis during this review are available upon request to corresponding author.

## Appendix 1

Form to extract data and relevance test.

**Table:**

Relevance test form		
Reading titles	Yes	No
Does the paper’s title address S/R and QoL?		
Is it explicit in the title whether the sample is composed of healthy individuals?		
*Reading abstracts and methods*		
Does the abstract address S/R and QoL?		
Is it explicit in the abstract whether the sample is composed of healthy individuals?		
Is it a primary study?		
Does it report a quantitative assessment of S/R and QoL measures?		
Are the instruments validated, recognized in the literature for the variables of interest?		
*Reading full texts*		
Is S/R and QoL the study’s central theme?		
Is the sample clearly composed of healthy adults?		
Is it a primary study?		
Are the instruments appropriate to the study’s criteria?		
Are the results adequately demonstrated?		
Is it an indexed periodical?		

## Abbreviations

QoL: Quality of life
E/R: Spirituality/religiousness
DUREL: Duke University Religious Index
EWBS: Existential Well-Being Scale
FACIT-Sp: The Functional Assessment of Chronic Illness Therapy–Spiritual Well-Being Scale
MHLC: Multidimensional Health Locus of Control Scale
RCOPE: Religious coping strategies
SAI: Spiritual attitude inventory
SIBS: Spiritual Involvement and Beliefs scale
SSRS: Spirituality Self-Rating Scale
STS: Spiritual Transcendence Scale
SCS: Spiritual Coping Strategy Scale
SWBQ-G: Spiritual well-being questionnaire Germany
SWBS: Spiritual Well-Being Scale
WHOQOL-SRPB: World Health Organization Quality of Life**-**Spirituality, Religiousness and Personal Beliefs
WHOQOL-bref: World Health Organization quality of life**-**brief

## References

[1] Fleck MP, Louzada S, Xavier M, Chachamovich E, Vieira G, Santos L, Pinzon V (2000). Aplicação da versão em português do instrumento abreviado de avaliação da qualidade de vida WHOQOL-bref. Rev Saude Publica.

[2] Seidl EMF, da Costa Zannon CML (2004). Qualidade de vida e saúde: aspectos conceituais e metodológicos. Cad Saude Publica.

[3] Moreno AB, Faerstein E, Werneck GL, Lopes CS, Chor D (2006). Propriedades psicométricas do Instrumento Abreviado de Avaliação de Qualidade de Vida da Organização Mundial da Saúde no Estudo Pró-Saúde. Cad Saude Publica.

[4] Haraldstad K, Wahl A, Andenæs R (2019). A systematic review of quality of life research in medicine and health sciences. Qual Life Res.

[5] Peres MFP, Kamei HH, Tobo PR, Lucchetti G (2018). Mechanisms behind religiosity and spirituality’s effect on mental health, quality of life and well-being. J Relig Health.

[6] Gobatto CA, Cristina T, de Araujo CF (2010). Coping religioso-espiritual: reflexões e perspectivas para a atuação do psicólogo em oncologia. Rev SBPH.

[7] Panzini RG, Bandeira DR (2007). Coping (enfrentamento) religioso/espiritual. Rev Psiq Clín.

[8] Baumstarck K, Alessandrini M, Hamidou Z, Auquier P, Leroy T, Boyer L (2017). Assessment of coping: a new french four-factor structure of the brief COPE inventory. Health Qual Life Outcomes.

[9] Zimpel RR, Panzini RG, Bandeira DR, Heldt E, Manfro GG, Fleck MP, da Rocha NS (2018). Can religious coping and depressive symptoms predict clinical outcome and quality of life in panic disorder? A Brazilian Longitudinal Study. J Nerv Ment Dis.

[10] Koenig HG (2012). Religion, spirituality, and health: the research and clinical implications. ISRN Psychiatry.

[11] Monod S, Brennan M, Rochat E, Martin E, Rochat S, Büla CJ (2011). Instruments measuring spirituality in clinical research: a systematic review. J Gen Intern Med.

[12] Marques LF, Aguiar APA (2014). Instrumentos de mensuração da religiosidade/espiritualidade (R/E) e seus construtos. Rev Pist Prax.

[13] Hall DE, Meador KG, Koenig HG (2008). Measuring religiousness in health research: review and critique. J Relig Health.

[14] Lucchetti G, Koenig HG, Pinsky I, Laranjeira R, Vallada H (2015). Spirituality or religiosity: is there any difference?. Rev Bras Psiquiatr.

[15] Lucchetti G, Granero Lucchetti AL, Vallada H (2013). Aferindo espiritualidade e religiosidade na pesquisa clínica: Uma revisão sistemática dos instrumentos disponíveis para a língua Portuguesa. Sao Paulo Med J.

[16] de Jager-Meezenbroek E, Garssen B, Van den Berg M, Van Dierendonck D, Visser A, Schaufeli WB (2010). Measuring spirituality as a universal human experience: a review of spirituality questionnaires. J Relig Health.

[17] Wang C-W, Chow AY, Chan CL (2017). The effects of life review interventions on spiritual well-being, psychological distress, and quality of life in patients with terminal or advanced cancer: a systematic review and meta-analysis of randomized controlled trials. Palliat Med.

[18] Lima S, Garrett C, Machado JC, Vilaça M, Pereira MG. Quality of life in patients with mild Alzheimer disease: the mediator role of mindfulness and spirituality. Aging Ment Health 2019; p. 1–8. 10.1080/13607863.2019.1650891 31411042

[19] Bonelli RM, Koenig HG (2013). Mental disorders, religion and spirituality 1990 to 2010: a systematic evidence-based review. J Relig Health.

[20] Cubukcu M (2018). Evaluation of quality of life in caregivers who are providing home care to cancer patients. Support Care Cancer.

[21] Colgrove LAA, Kim Y, Thompson N (2007). The effect of spirituality and gender on the quality of life of spousal caregivers of cancer survivors. Ann Behav Med.

[22] Desbiens JF, Fillion L (2007). Coping strategies, emotional outcomes and spiritual quality of life in palliative care nurses. Int J Palliat Nurs.

[23] Ali J, Marhemat F, Sara J, Hamid H (2015). The relationship between spiritual well-being and quality of life among elderly people. Holist Nurs Pract.

[24] Zimmer Z, Jagger C, Chiu CT, Ofstedal MB, Rojo F, Saito Y (2016). Spirituality, religiosity, aging and health in global perspective: a review. SSM Popul Heal.

[25] Counted V, Possamai A, Meade T (2018). Relational spirituality and quality of life 2007 to 2017: An integrative research review. Health Qual Life Outcomes.

[26] de Bernardin-Gonçalves JP, Lucchetti G, Menezes PR, Vallada H (2017). Complementary religious and spiritual interventions in physical health and quality of life: a systematic review of randomized controlled clinical trials. PLOS ONE.

[27] Zhang KC, Hui CH, Lam J, Lau EYY, Cheung S, Mok DSY (2014). Personal spiritual values and quality of life: evidence from Chinese college students. J Relig Health.

[28] Pillay N, Ramlall S, Burns JK (2016). Spirituality, depression and quality of life in medical students in KwaZulu-Natal. South African J Psychiatry.

[29] Deb S, Strodl E (2018). Quality of Life and Spirituality in Indian University Students. Appl Res Qual Life.

[30] Krägeloh CU, Henning MA, Billington R, Hawken SJ (2015). The relationship between quality of life and spirituality, religiousness, and personal beliefs of medical students. Acad Psychiatry.

[31] Moher D, Liberati A, Tetzlaff J, Altman DG (2009). Preferred reporting items for systematic reviews and meta-analyses: the PRISMA statement. Ann Intern Med.

[32] Booth A, Clarke M, Dooley G, Ghersi D, Moher D, Petticrew M, Stewart L (2012). The nuts and bolts of PROSPERO: an international prospective register of systematic reviews. Syst Rev.

[33] World Health Organization. international statistical classification of diseases and related health problems ICD-10: instruction manual. Geneva: World Health Organization: 2016, 10th revision 5a ed; 2:1–252. Available from: https://icd.who.int/browse10/Content/statichtml/ICD10Volume2_en_2016.pdf.

[34] World Health Organization. Databases—regional health observatory. World Health Statistics: monitoring health for the SDGs. Geneva: World Health Organization: 2016. Available from: https://www.who.int/gho/publications/world_health_statistics/2016/en/.

[35] Landis JR, Koch GG (1977). The measurement of observer agreement for categorical data. Biometrics.

[36] Joanna Briggs Institute. The Joanna Briggs Institute Reviewers’ manual: 2014 edition . Joanna Briggs Institute: Austrália, 2014.

[37] Hsu PH-C, Krägeloh CU, Shepherd D, Billington R (2009). Religion/spirituality and quality of life of international tertiary students in New Zealand: an exploratory study. Ment Heal Relig Cult.

[38] Casu G, Ulivi G, Zaia V, Fernandes Martins MDC, Parente Barbosa C, Gremigni P (2018). Spirituality, infertility-related stress, and quality of life in Brazilian infertile couples: analysis using the actor-partner interdependence mediation model. Res Nurs Heal.

[39] Dadkhahtehrani T, Momenyan S, Heidari S, Momenyan N (2018). Association between the religious coping of infertile people with their own quality of life and their spouses’: a correlation study in Iranian infertile couples. Iran J Nurs Midwifery Res.

[40] Gonçalves LM, Tsuge MLT, Borghi VS, Miranda FP, de Assis Sales AP, Lucchetti ALG, Lucchetti G (2018). Spirituality, Religiosity, Quality of Life and Mental Health Among Pantaneiros: A Study Involving a Vulnerable Population in Pantanal Wetlands. Brazil. J Relig Health.

[41] Chai PPM, Krägeloh CU, Shepherd D, Billington R (2012). Stress and quality of life in international and domestic university students: Cultural differences in the use of religious coping. Ment Heal Relig Cult.

[42] Felicilda-Reynaldo RFD, Cruz JP, Papathanasiou IV, Helen Shaji JC, Kamau SM, Adams KA, Valdez GFD (2019). Quality of life and the predictive roles of religiosity and spiritual coping among nursing students: a multi-country study. J Relig Health.

[43] Lau WWF, Hui CH, Lam J, Lau EYY, Cheung S-F (2015). The relationship between spirituality and quality of life among university students: an autoregressive cross-lagged panel analysis. High Educ.

[44] Frenk J, Gómez-Dantés O (2014). Designing a framework for the concept of health. J Public Health Policy.

[45] Goncąlves JPB, Lucchetti G, Menezes PR, Vallada H (2015). Religious and spiritual interventions in mental health care: a systematic review and meta-analysis of randomized controlled clinical trials. Psychol Med.

[46] Tam W, Lo K, Pacheco J (2019). Prevalence of depressive symptoms among medical students: overview of systematic reviews. Med Educ.

[47] Storrie K, Ahern K, Tuckett A (2010). A systematic review: students with mental health problems—a growing problem. Int J Nurs Pract.

[48] Vitorino LM, Lucchetti G, Leão FC, Vallada H (2018). Peres MFP (2018) The association between spirituality and religiousness and mental health. Sci Rep.

[49] Ashouri FP, Hamadiyan H, Nafisi M, Parvizpanah A (2016). The relationships between religion/spirituality and mental and physical health: a review. Int Electron J Med.

[50] Barroso SM, de Oliveira NR, de Andrade VS (2019). Solidão e Depressão: Relações com Características Pessoais e Hábitos de Vida em Universitários. Psicol Teor e Pesqui.

[51] Rhodes JL (2014). Loneliness: how superficial relationships, identity gaps, and social support contribute to feelings of loneliness at pepperdine university. J Commun Res.

[52] Momeni K, Moradi S, Dinei S, Shahrestani A, Dinei M, Mohammadi F, Dabirian M (2017). The relationship between quality of life, spirituality, and resilience and suicidal thoughts in students of Razi University. Ann Trop Med Public Heal.

